# Molecular Epidemiology of Neonatal-Associated Staphylococcus haemolyticus Reveals Endemic Outbreak

**DOI:** 10.1128/spectrum.02452-22

**Published:** 2022-10-31

**Authors:** Ronja Westberg, Marc Stegger, Bo Söderquist

**Affiliations:** a School of Medical Sciences, Faculty of Medicine and Health, Örebro University, Örebro, Sweden; b Department of Bacteria, Parasites, and Fungi, Statens Serum Institut, Copenhagen, Denmark; University of Calgary

**Keywords:** whole-genome sequencing, molecular epidemiology, neonatal sepsis, hospital-acquired infections, *Staphylococcus haemolyticus*, coagulase-negative *staphylococcus*

## Abstract

Staphylococcus haemolyticus is a major cause of late-onset sepsis in neonates, and endemic clones are often multidrug-resistant. The bacteria can also act as a genetic reservoir for more pathogenic bacteria. Molecular epidemiology is important in understanding bacterial pathogenicity and preventing infection. To describe the molecular epidemiology of *S. haemolyticus* isolated from neonatal blood cultures at a Swedish neonatal intensive care unit (NICU) over 4 decades, including antibiotic resistance genes (ARGs), virulence factors, and comparison to international isolates. Isolates were whole-genome sequenced, and single nucleotide polymorphisms in the core genome were used to map the relatedness. The occurrence of previously described ARGs and virulence genes were investigated. Disc diffusion and gradient tests were used to determine phenotypic resistance. The results revealed a clonal outbreak of *S. haemolyticus* at this NICU during the 1990s. Multidrug resistance was present in 28 (82%) of all isolates and concomitant resistance to aminoglycoside and methicillin occurred in 27 (79%). No isolates were vancomycin resistant. Genes encoding ARGs and virulence factors occurred frequently. The isolates in the outbreak were more homogenous in their genotypic and phenotypic patterns. Genotypic and phenotypic resistance combinations were consistent. Pathogenic traits previously described in *S. haemolyticus* occurred frequently in the present isolates, perhaps due to the hospital selection pressure resulting in epidemiological success. The clonal outbreak revealed by this study emphasizes the importance of adhering to hygiene procedures in order to prevent future endemic outbreaks.

**IMPORTANCE** This study investigated the relatedness of Staphylococcus haemolyticus isolated from neonatal blood and revealed a clonal outbreak in the 1990s at a Swedish neonatal intensive care unit. The outbreak clone has earlier been isolated in Japan and Norway. Virulence and antibiotic resistance genes previously associated with clinical *S. haemolyticus* were frequently occuring in the present study as well. The majority of the isolates were multidrug-resistant. These traits should be considered important for *S. haemolyticus* epidemiological success and are probably caused by the hospital selection pressure. Thus, this study emphasizes the importance of restrictive antibiotic use and following the hygiene procedures, to prevent further antibiotic resistance spread and future endemic outbreaks.

## INTRODUCTION

Staphylococcus haemolyticus is a coagulase-negative staphylococcus (CoNS) and a normal part of the human microbiome (1, 2). This species is clinically relevant since it acts as an opportunistic pathogen and is often multidrug-resistant (MDR), defined as resistance to three or more antibiotic groups (3–5). Due to its biofilm-forming ability, it is predisposed to enter the circulation by colonization of indwelling medical devices such as catheters, prostheses, and other instruments, and can therefore cause nosocomial infections in the most critically ill patients (6, 7).

Preterm neonates are an important patient group in terms of invasive nosocomial CoNS infections, since CoNS is the major cause of late onset sepsis (LOS, age >72h) (8–12). LOS has a reported global incidence of nearly 1,000 per 100,000 births and a mortality rate of >16% (13), and confers an increased risk of permanent neurological impairment (14). Improved neonatal care has increased survival rates of preterm and very low birth weight neonates (<1,500 g), which are the greatest risk factors for LOS (10–12, 15). *S. haemolyticus* present at neonatal intensive care units (NICU) are often endemic clones (8, 16–18).

A comparison of commensal and clinical *S. haemolyticus* isolates found clear differences in genetic determinants and genotypes associated with pathogenicity and success in the hospital environment (19). A previous study of another CoNS, Staphylococcus capitis, showed neonatal-specific pathogenicity traits coinciding with the establishment of NICUs in the 1960s, creating the neonatal specific clone NRSC-A (20). These pathogenic traits in CoNS can move horizontally between species, especially those more closely related, and *S. haemolyticus* is therefore suggested to act as a gene reservoir for more pathogenic bacteria, such as Staphylococcus aureus (5, 21–25). Whole-genome sequencing (WGS) studies on *S. haemolyticus* suggests that its genome is highly variable, and that new resistance markers and virulence factors can be acquired by horizontal gene transfer (HGT) and point mutations. (3, 4, 26–28).

The present study aimed to describe the molecular epidemiology of *S. haemolyticus* isolated from neonatal blood cultures at a Swedish NICU over 4 decades, including the presence of antibiotic resistance genes (ARG), virulence factors, and comparison to international isolates.

## RESULTS

### Phylogeny and population structure.

Of the 34 neonatal blood cultures that were available for analysis, all passed the WGS-based quality control.

**FIG 1:**
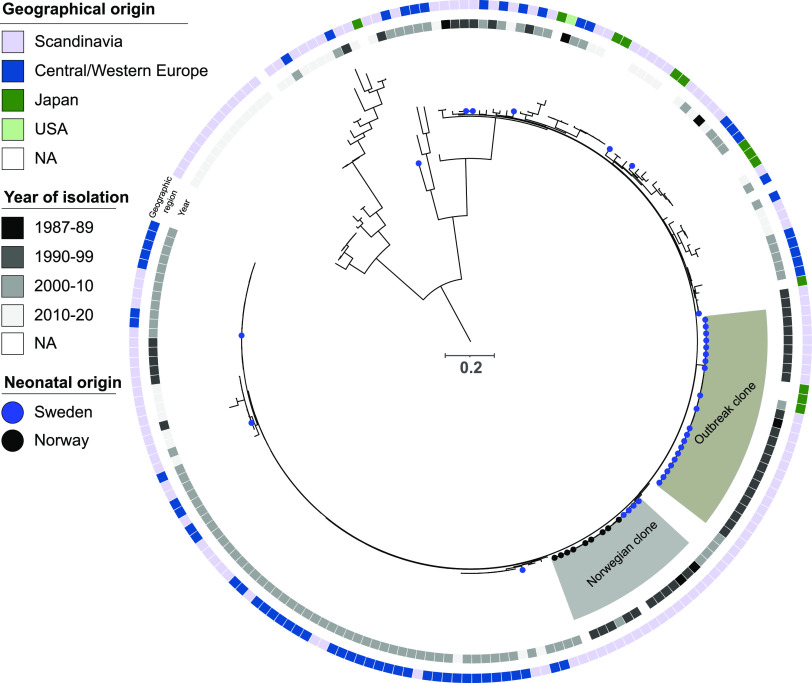
Midpoint rooted phylogeny of the 34 *S. haemolyticus* neonatal isolates obtained between 1980 and 2020 in Örebro, Sweden, along with publicly available genomic data (*n* = 175), based on 78,373 SNPs in the conserved core genome. The Swedish neonatal isolates are marked with a blue circle. Neonatal isolates with other geographical origin are marked with a black circle. The Swedish outbreak clone (ST2) and the cluster of 12 Norwegian isolates with four isolates from Sweden are highlighted in gray. The inner circle displays isolation year, and the outer circle the geographical origin.

**FIG 2:**
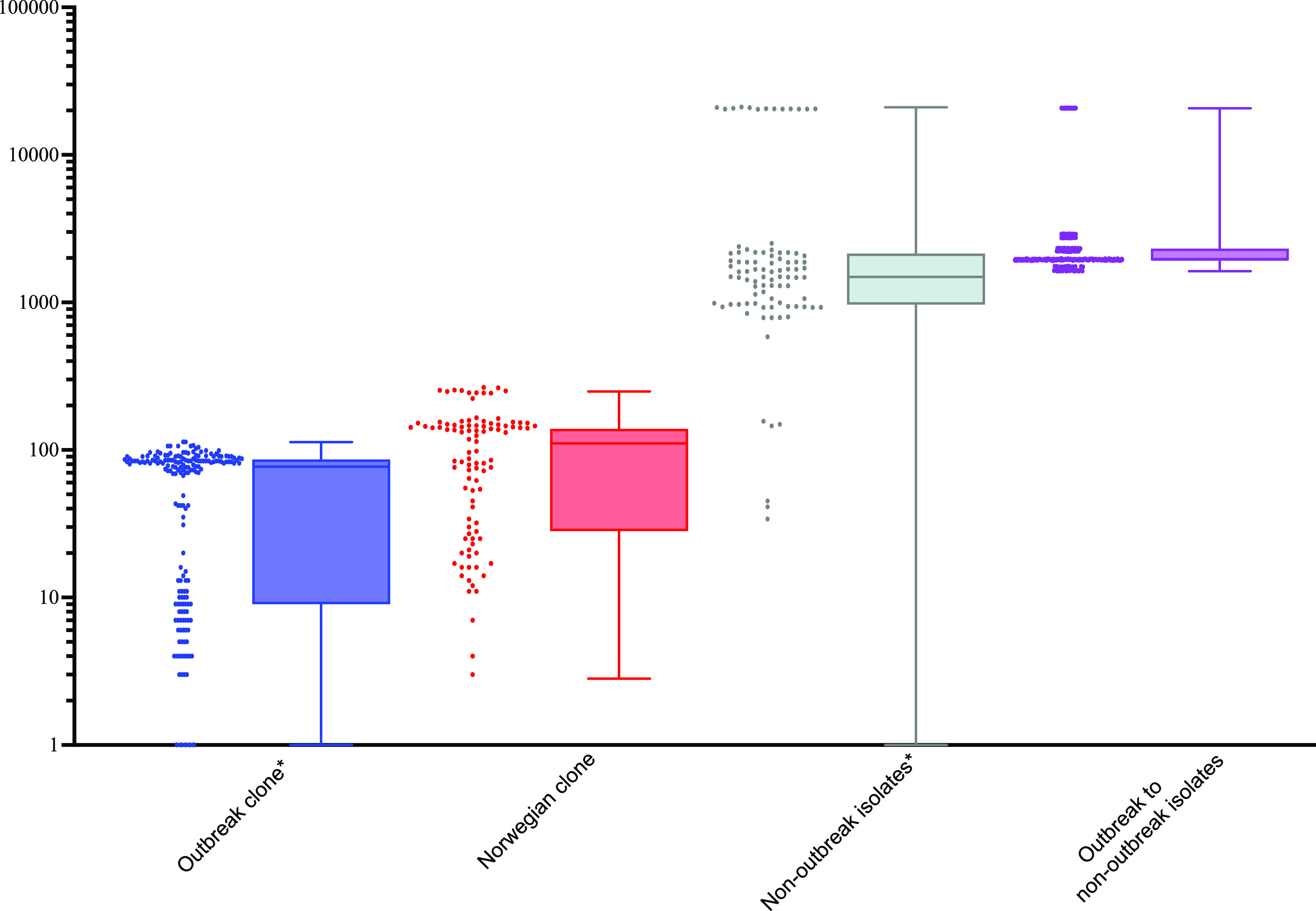
Pairwise SNP distance between *S. haemolyticus* bacterial isolates within the outbreak clone (*n* = 20), within the non-outbreak isolates (*n* = 14), within the Norweigan-dominated clade (*n* = 16; four from Örebro and 12 from Norway), and the outbreak clone compared to non-outbreak isolates. Y-axis is logarithmic and * indicates the presence of pairwise SNP distance of zero, four within the outbreak clone and one within the non-outbreak isolates.

The relatedness of these isolates was examined in comparison with 175 international *S. haemolyticus* isolates, showing a total of 78,373 SNPs within a conserved core genome of 58.8%. This phylogenetic analysis showed that the Swedish isolates did not belong to one single monophyletic group; instead, there was one cluster consisting of 20 ST 2 isolates with pairwise SNP distances of between 0 and 113 bp, and 14 sporadic cases scattered throughout the phylogeny (Fig. 1) with a variety of different or novel STs. The 20 isolates that clustered are here referred to as the outbreak clone and the other Swedish isolates as non-outbreak isolates. Three isolates from Japan and three from Norway also clustered as part of the outbreak clone with pairwise SNP distances of 59 to 117 bp compared to the Swedish isolates in that cluster but these were not isolated from neonates. When the Swedish outbreak clade isolates were compared to the Swedish non-outbreak isolates, the pairwise SNP distances in the core genome were between 1,626 and 20,685 bp (Fig. 2).

Four of the Swedish non-outbreak isolates clustered with 12 Norwegian isolates, with pairwise SNP distances ranging from 3 to 265 bp between all isolates in that cluster (Fig. 2). All isolates, except two with unknown sampling year, within this cluster with known sampling year were isolated between 1991 and 2002. Nine of the Norwegian isolates (75%) were from a neonatal unit and three were of unknown origin (Fig. 1).

### Phenotypical antibiotic resistance.

Phenotypic antibiotic susceptibility testing on the Swedish isolates showed MDR in 18 (90%) of the outbreak isolates and 10 (71%) of the non-outbreak isolates, for a total of 28 (82%). Comparing outbreak to non-outbreak isolates, the outbreak isolates were more frequently resistant to aminoglycoside (gentamicin, *P* = 0.022), and the non-outbreak isolates were more frequently resistant to fluoroquinolone (norfloxacin, *P* = 0.022), and fusidic acid (*P* = 0.022). No statistically significant differences were found between the two groups for resistance to methicillin (cefoxitin), lincosamide (clindamycin), macrolide (erythromycin), or trimethoprim-sulfamethoxazole (*P* > 0.05; Table 1). No isolates were resistant to glycopeptides (vancomycin), oxazolidinones (linezolid), and rifamycins (rifampicin). Eleven different resistance patterns were found in total in the 34 isolates. The most prevalent of these was resistance to aminoglycoside, methicillin, and macrolide (Table S1), which was seen in 16 (80%) of the outbreak clade isolates and in five (36%) of the other Swedish isolates. Contemporary resistance to aminoglycoside and methicillin occurred in all of the outbreak clade isolates and in 50% of the non-outbreak isolates.

**TABLE 1:** Frequency of phenotypic antibiotic resistance for the 34 *S. haemolyticus* isolates

Antibiotic class	Substance	Outbreak clone n (%)	Non-outbreak isolates n (%)	*P* value^*a*^
Aminoglycoside	Gentamicin	20 (100)	10 (71)	0.022
Methicillin	Cefoxitin	20 (100)	11 (79)	0.061
β-lactam	Penicillin G	20 (100)	14 (100)	1
Fluoroquinolones	Norfloxacin	0	4 (29)	0.022
Fusidic acid	Fusidic acid	0	4 (29)	0.022
Glycopeptide	Vancomycin	0	0	1
Lincosamide	Clindamycin	0	3 (21)	0.061
Lipopeptide	Daptomycin	0	0	1
Macrolide	Erytromycin	18 (90)	9 (64)	0.097
Oxazolidinones	Linezolid	0	0	1
Rifamycin	Rifampicin	0	0	1
Trimethoprim-sulfamethoxazole	Trimethoprim-sulfamethoxazole	2 (10)	3 (21)	0.627
MDR		20 (100)	10 (71)	0.022

a *P*-values calculated with Fisher’s exact test, comparing the outbreak clone to the non-outbreak isolates.

### Genotypical antibiotic resistance determinants.

Genotypically, 33 isolates (97%) were MDR. Comparing outbreak to non-outbreak isolates, the resistance results were: 0 versus 4 (0% versus 29%; *P* = 0.022) for chloramphenicol (*cat (pC221)*), 0 versus 4 (0% versus 29%; *P* = 0.022) for fusidic acid (*fusB*), 18 versus 7 (90% versus 50%; *P* = 0.017) for macrolide (*mphC*), 18 versus 7 (90% versus 50%; *P* = 0.017) for macrolide (*msrA*), and 0 versus 4 (0% versus 29%; *P* = 0.022) for tetracycline (*tet*[K]). No statistically significant differences between the two groups were found for the other resistance genes (Table S2). Twelve different resistance patterns were found in total. The most prevalent combination was resistance to aminoglycoside *(aac[6′]-aph[2′'])*, β-lactam (*blaZ*), disinfectant (*qacA*), macrolide (*mphC*), macrolide (*msrA*), and methicillin (*mecA*), which was seen in 15 (75%) of the outbreak clade isolates and the 4 (29%) of the non-outbreak isolates that clustered with the Norwegian isolates (Table S1). Investigation of the SCC element comprised by isolates in the outbreak clone revealed no previously known *ccr* genes and thus point to a novel SCC*mec* type.

### Genotypical and phenotypical concordance for antibiotic resistance.

The phenotypic and genotypic marginal homogeneity were: *P* = 1 for aminoglycosides (gentamicin compared to *aac(6′)-aph(2′')*), *P* = 1 for β-lactams (penicillin G compared to *blaZ*), *P* = 1 for methicillin (cefoxitin compared to *mecA*), *P* = 1 for fusidic acid (fusidic acid compared to *fusB* and *fusC*), *P* = 1 for macrolides (erythromycin compared to *mphC*, *ermA*, *msrA*, *vga[A]LC*, and *ermC*), *P* = 1 for lincosamide (clindamycin compared to *lnuA*), and *P* = 0.5 for trimethoprim-sulfamethoxazole (trimethoprim-sulfamethoxazole compared to *dfrG*).

Two isolates carried aminoglycoside resistance genes not specific for gentamicin; one had *aadD* and one *ant(*9*)-Ia*. The only macrolide resistance gene encountered alone was *ermA*, since *mphC* and *msrA* always occurred together and *vga(A)LC* and *ermC* were always accompanied by another macrolide resistance gene. All combinations caused phenotypic resistance. The fusidic acid resistance gene *fusC* was codetected in all isolates carrying the *fusB* gene.

### Virulence.

All outbreak isolates carried all (20) previously determined virulence genes associated with nosocomial infections (Table S2). All 20 virulence genes were present in the non-outbreak isolates as well, but they were more diverse, showing 8 different patterns. Two of the non-outbreak isolates also contained all the virulence genes, but they were not related in the phylogeny (Table 2).

## DISCUSSION

The burden of neonatal LOS is still an issue. Preterm and very low birth weight neonates constitute a vulnerable patient group in this respect, and the increased survival that has come with improved care means that their numbers are growing. Molecular typing of LOS pathogens is important in monitoring the epidemiology, eliminating established hospital clones, and preventing the emergence of any future such clones (29, 30). In this study, molecular epidemiology of *S. haemolyticus* neonatal blood culture isolates revealed that 20 of 34 isolates belonged to an epidemiological outbreak clone in the Örebro NICU during the 1990s, while the remaining 14 were diverse compared to the international collection. The majority of Swedish isolates showed resistance to commonly used antimicrobial agents, as well as genes encoding resistance to disinfectants and proteins involved in adhesion and human immune defense escape.

The outbreak clone described in this study also included three isolates from Norway and three from Japan, all from unspecified human infections or of unknown origin. A second cluster was observed involving 12 Norwegian and four of the Swedish non-outbreak isolates, all from the same time period and from neonates when information was available. The earliest identified outbreak isolate was from Norway, 1989. In the second, Norwegian cluster, the earliest identified isolate was from Sweden, 1987 (19). This indicates survival in the hospital environment and the ability to spread via human travel and international employment. It is possible that the second cluster is a neonatal niche of *S. haemolyticus*, as suggested for NRSC-A (20), but further studies are needed to confirm or disapprove this.

Comparison between commensal and invasive *S. haemolyticus* pathogenicity traits have been done before (19), but, to our knowledge, no studies have investigated neonatal *S. haemolyticus* specific traits, as with NRSC-A (20). The present study investigated the occurrence of genes reported by Pain et al. (19) to be the most prevalent in clinical isolates. Our findings were clearly in concordance with those of the earlier study (19), as all outbreak isolates harbored all analyzed virulence genes. These genes therefore also seem to be of importance for the epidemiological success of the clone circulating at the Swedish NICU.

Acquisition of MDR is an expected adaption of nosocomial pathogens, giving advantage in an environment with high drug selection pressure (19). Ward-specific CoNS resistance patterns have previously been described (20, 31, 32). In concordance with these previous studies (20, 31, 32), the majority of isolates in the present study were MDR and resistant to both aminoglycoside and β-lactam antibiotics, which are the first line treatment for LOS in Sweden (33). This indicates adaption to selection pressure. However, the high rates of macrolide resistance cannot be explained by the antibiotics used in NICUs and, unlike the situation for β-lactams and aminoglycosides, this has not been described previously as a neonatal-specific CoNS attribute (20). Although macrolide resistance has been previously described as being closely related to *S. haemolyticus* (19) and also found in neonatal units in several CoNS (17), the macrolide resistance indicates circulation in other wards. It remains to be discovered whether this has any role in the neonatal epidemic success. Even the *S. capitis* neonatal clone NRSC-A is not entirely neonatal-specific, as it has caused prosthetic joint infections (34).

If resistance to the first line treatment is present, vancomycin is generally used. No isolates of Swedish origin in the study were resistant to this antibiotic. Nonetheless, cases have been reported, and this could constitute a future problem (35, 36). With resistance to methicillin and aminoglycosides, the use of vancomycin might increase, which could both result in vancomycin-resistant *S. haemolyticus* and create spread to other staphylococci via HGT. Vancomycin resistance is also suggested as a major driver of neonatal clone success in *S. capitis*, illustrating how extensive use of antibiotics can be a driver of MDR evolution from otherwise commensal drug-susceptible bacteria; these resistant bacteria are then able to spread worldwide and cause infections among highly vulnerable patient groups (20, 34). Similar scenarios have been described in other CoNS, such as Staphylococcus epidermidis, with global spread of nosocomial MDR due to selection pressure (37, 38).

MDR *S. haemolyticus* in the neonatal skin flora has been shown to increase during hospitalization (32), and has also been reported to colonize surfaces in the hospital environment (39, 40). Neonatal-specific *S. capitis* clades are known to colonize the NICU environment, especially incubators (41). However, CoNS bacteremia can be significantly reduced with implementation of improved hygiene standards (9), and so it is of great importance for hospital personnel to maintain hygiene routines.

Genotypic and phenotypic resistance patterns were consistent. Well-established resistance determinants were identified for β-lactams (*blaZ)*, methicillin (*mecA)*, aminoglycosides (*aac[6′]-aph(2′′)*), macrolides (*mphC*, *ermA*, *msrA*, *vga[A]LC*, and *ermC*), clindamycin (*lnuA*), and trimethoprim-sulfamethoxazole (*dfrG*). Neither the macrolide resistance genes *mphC*, *msrA*, *vga(A)LC*, and *ermC* nor the fusidic acid resistance gene *fusC* occurred as single genes for the corresponding phenotypic resistance, and so it is impossible to say if the phenotypic resistance was generated by these genes, their accompanying genes, or both.

Another gene frequently occurring in the *S. haemolyticus* isolates in this study was *qacA*, which encodes an efflux pump that causes decreased susceptibility to chlorhexidine, and is also connected to other resistance mechanisms (42, 43). The gene is related to staphylococcal infective isolates (19, 43), and the *qacA* in *S. haemolyticus* has been reported to have a high similarity to the *qacA* found in Staphylococcus aureus (42). Consequently, *qacA* could be considered both as a *S. haemolyticus* pathogenic trait and as an indicator that *S. haemolyticus* acts via HGT as a gene reservoir for more pathogenic bacteria.

This is the first molecular epidemiological study of *S. haemolyticus* on a neonatal ward in Sweden. The epidemiology of the neonatal ward in Örebro is described reliably, since all isolates in the present study were collected from the same unit with the same indications during a long period of time. There are, however, some limitations to this study. First, it is not possible to say whether the traits in the investigated isolates are neonatal-specific or not, only that the attributes previously described as overrepresented in clinical isolates also were present in these neonatal isolates. Second, there are other suggested important virulence determinants for *S. haemolyticus*, such as biofilm formation, insertion sequences, plasmids, and phenol-soluble modulins (19). Third, it is still not known how important macrolide resistance is, where it comes from, and whether this resistance is connected to other virulence factors important for survival in the hospital environment and/or NICUs.

For a deeper understanding of the pathogenic traits of *S. haemolyticus*, there are several comparisons that would be of interest. First, comparing clinical isolates to commensal isolates would allow continued investigation of potential invasive traits. This could specify markers for differentiation between contamination and infection, and also between different pathogenicities. In the long run, this could lead to more restrictive antibiotic use and new, more specific, treatments. Second, comparing neonatal clinical isolates to other clinical isolates (such as those related to indwelling medical devices) from all over the world would make it possible to see whether there are neonatal-specific attributes and how widespread the different identified clones are. This could be used to trace invasive isolates and eliminate them from the hospital environment. Third, comparing colonization patterns of premature neonates to those from the usual maternity ward would bring improved understanding of when, where, and why infection appears, and thus facilitate its prevention.

### Conclusions.

This study reveals a clonal outbreak of *S. haemolyticus* at a NICU, which emphasizes the importance of adhering to hygiene procedures in order to prevent future endemic outbreaks. Previously described pathological traits of *S. haemolyticus* occurred frequently in the present isolates, perhaps due to hospital selection pressure resulting in epidemiological success. The majority of isolates were resistant to the first line treatment for LOS (aminoglycoside and β-lactams). Continuous molecular typing is important in order to monitor the epidemiology, to eliminate established hospital clones, and to prevent the emergence of other such clones in the future. Further studies are needed to connect specific invasive traits to the epidemiological success of *S. haemolyticus*.

## MATERIALS AND METHODS

### Bacterial isolates.

*S. haemolyticus* isolates were obtained from blood cultures from newborns ≤28 days of age with clinical signs of sepsis in the NICU at the Department of Paediatrics, Örebro University Hospital, Örebro, Sweden, between 1980 and 2020. One sample from each patient were preserved as pure cultures at −80°C in preservation medium consisting of Trypticase soy broth supplemented with 0.3% yeast extract (BD Diagnostic Systems, Sparks, MD, USA) and 29% horse serum (Håtunalab AB, Håtuna, Sweden) according to routine procedures. Of the 38 detected *S. haemolyticus* isolates from individual newborns, 34 were available for analyses. For comparison to international circulating strains, 175 previously described *S. haemolyticus* isolates with known origin were included (2, 44).

### Genome sequencing.

The 34 *S. haemolyticus* isolates were incubated overnight at 36°C on blood agar plates (SSI Diagnostica, Denmark), and then their DNA was purified using the Roche MagNA Pure 96 system (F. Hoffman-La Roche Ltd., Basel, Switzerland). DNA was quantified using the Qubit fluorometer (Invitrogen, Waltham, MA, USA), followed by library preparation using the Nextera XT DNA Library Prep kit (Illumina Inc., San Diego, CA, USA) according to the manufacturer’s protocol. Sequencing was performed on a NextSeq 550 platform (Illumina Inc., San Diego, CA, USA) to obtain paired-end reads using a 300-cycle kit. The sequencing data were subjected to quality control using bifrost (https://github.com/ssi-dk/bifrost) to ensure adequate (>50-fold) sequencing depth, and tested for contamination of all isolates prior to assembly using SPAdes v3.9.0 (45).

### Phylogeny.

Single nucleotide polymorphisms (SNPs) in the core genome were used to map the relatedness of the *S. haemolyticus* isolates. The raw sequence data were aligned against the chromosome of *S. haemolyticus* reference strain 12b (GenBank accession number CP071505). Identification of SNPs was performed using NASP v.1.2.1 (46), with removal of duplicated regions using NUCmer (47). All positions with less than 10-fold sequencing depth and 90% unambiguous variant calls for any isolate were excluded. Phylogenetic reconstruction was performed using IQ-TREE v1.6.12 (48), with ModelFinder as implemented in IQ-TREE. Phylogenetic robustness was assessed with bootstrap analysis using 100 replicates. Finally, the phylogenies were visualized and annotated using iTol v6.4.1 (49).

### Resistance and virulence and typing.

ARGs were detected in raw sequence data using ARIBA (50) with default settings with gene presence cutoff at >90% length and sequence similarity using the ResFinder database (51), accessed September 23, 2021. To identify virulence genes which may underlie the invasive success of *S. haemolyticus*, 20 virulence genes previously identified and related to clinical isolates (19) were chosen and were similarly assessed using ARIBA with the raw sequencing data. Sequence types (STs) of the isolates were obtained using mlst (https://github.com/tseemann/mlst), whereas Staphylococcal Cassette Chromosome *mec* variants were detected using SCC*mec*Finder (https://cge.cbs.dtu.dk/services/SCCmecFinder/).

### Antibiotic susceptibility testing.

Antibiotic susceptibility for the available 34 *S. haemolyticus* isolates was determined by disc diffusion, performed and interpreted according to European Committee on Antimicrobial Susceptibility Testing (EUCAST) guidelines (http://www.eucast.org, clinical breakpoints v 11.0). The antibiotics tested were linezolid (10 μg), cefoxitin (30 μg), fusidic acid (10 μg), clindamycin (2 μg), erythromycin (15 μg), gentamicin (10 μg), rifampicin (5 μg), trimethoprim–sulfamethoxazole (25 μg), and norfloxacin (10 μg) (all discs from Oxoid, Basingstoke, Hampshire, England). Vancomycin and daptomycin MIC values were determined using Etest (bioMérieux, Marcy l’Etoile, France) according to the manufacturer’s instructions.

### Ethics.

The bacterial isolates originated from blood cultures collected according to clinical routine. These isolates were subcultured, and so no human biological material was stored. Pure clinical isolates were preserved as per clinical routine. No patient data concerning the isolates was available. Bacterial research is not included in the Swedish Act concerning the ethical review of research involving humans (2003:460).

### Statistics.

Fisher’s exact test was used to analyze the prevalence of antibiotic resistance phenotype, ARGs, and virulence genes. McNemar’s test was used to examine marginal homogeneity between phenotypic and genotypic antibiotic resistance patterns. All analyses were performed in version 28.0 of IBM SPSS Statistics (IBM Corp., USA). *P*-values <0.05 were considered statistically significant.

### Data availability.

All genomic data of the Swedish isolates used for this study have been deposited at the European Nucleotide Archive (https://www.ebi.ac.uk/ena/browser/home) with BioProject number PRJEB56240.
