# Supplementary material for: Molecular Epidemiology of Neonatal-Associated Staphylococcus haemolyticus Reveals Endemic Outbreak
Source: Microbiol Spectr. 2022 Oct 31;10(6):e02452-22. doi: 10.1128/spectrum.02452-22 (PMC9769988; doi:10.1128/spectrum.02452-22)
Supplement: Supplemental file 1 — Tables S1 and S2. Download spectrum.02452-22-s0001.pdf, PDF file, 0.2 MB [file spectrum.02452-22-s0001.pdf]

Table S1. All investigated resistance- and virulence genes in the 34 S. haemolyticus *isolates. P-values calculated with Fisher's exact test, comparing the outbreak clone to the non-outbreak isolates. Virulence genes previously associated with clinical* S. haemolyticus *isolates (1).*

Gene	Function/resistance	Outbreak clone n (%)	Non-outbreak isolates n (%)	p-value
**Resistance genes**				
aac(6')-aph(2'')	aminoglycoside	20 (100)	10 (71)	0.022
aadD	aminoglycoside	0	1 (7)	0.412
ant(9)-Ia	aminoglycoside	0	1 (7)	0.412
aph(3')-III	aminoglycoside	0	0	1
str	aminoglycoside	0	0	1
qacB	antiseptic	0	0	1
blaZ	β-lactam	20 (100)	14 (100)	1
cat (pC221)	chloramphenicol	0	4 (29)	0.022
qacA	disinfectant	17 (85)	12 (86)	1
fusB	fusidic acid	0	4 (29)	0.022
fusC	fusidic acid	0	1 (7)	0.412

lnuA	lincosamide	0	2 (14)	0.162
lsaA	lincosamides and streptogramin a	0	0	1
ermA	macrolide	0	1 (7)	0.412
ermC	macrolide	0	1 (7)	0.412
mphC	macrolide	18 (90)	7 (50)	0.017
vga(A)LC	macrolide	0	2 (14)	0,162
msrA	macrolide	18 (90)	7 (50)	0.017
mecA	methicillin	20 (100)	11 (79)	0.061
***tet*(K)**	tetracycline	0	4 (29)	0.022
***tet*(M)**	tetracycline	0	1 (7)	0.412
dfrG	trimethoprim	0	3 (21)	0.061
**MDR**		20 (100)	13 (93)	0.412

Virulence genes

ugpQ	Glycerophosphoryl diester phosphodiesterase	20 (100)	11 (79)	0.061
paaZ	Domain protein	20 (100)	11 (79)	0.061

mecA	Methicillin resistance	20 (100)	11 (79)	0.061
SH0155_NA	MFS transporter	20 (100)	14 (100)	1
nikA	Nickel-binding periplasmic protein	20 (100)	14 (100)	1
secA2	Protein translocase subunit seca2	20 (100)	13 (93)	0.412
fabG	Fabg	20 (100)	13 (93)	0.412
yjjG	Pyrimidine 5'-nucleotidase yjjg	20 (100)	14 (100)	1
ansA	Putative L-asparaginase	20 (100)	14 (100)	1
aacA-aphD	Bifunctional AAC/APH	20 (100)	10 (71)	0.022
SH1612_NA	Acetyltransferase GNAT	20 (100)	10 (71)	0.022
blaI	Penicillinase repressor	20 (100)	13 (93)	0.412
blaR1	Regulatory protein blar1	20 (100)	12 (86)	0.162
blaZ	B-lactamase	20 (100)	14 (100)	1
SH2156_NA	Ferrichrome ABC transporter truncated	20 (100)	13 (93)	0.412
tagE	Truncated glycosyltransferase	20 (100)	13 (93)	0.412

folB	Dihydroneopterin aldolase	20 (100)	8 (57)	0.002
folP	Dihydropteroate synthase	20 (100)	9 (64)	0.007
SH2607_NA	ATP-binding protein (plasmid)	20 (100)	4 (29)	<0.001
sraP	Serine-rich adhesin for platelets	20 (100)	13 (93)	0.412

Table S2. Frequency of combinations of antibiotic resistance phenotype, genotype and virulence seen in the 34 S. haemolyticus *isolates.*

Combination	Antibiotic resistance phenotype	Outbreak clone n (%)	Non-outbreak isolates n (%)
**1**	Aminoglycoside, Methicillin, β-lactam, Macrolide	16 (80)	5 (36)
**2**	Aminoglycoside, Methicillin, β-lactam	2 (10)	0 (0)
**3**	Aminoglycoside, Methicillin, β-lactam, Macrolide, Trimethoprim-sulfamethoxazole	2 (10)	0 (0)
**4**	Fusidic acid, β-lactam	0 (0)	1 (7)
**5**	Aminoglycoside, Methicillin, β-lactam, Fluoroquinolone	0	1 (7)
**6**	Aminoglycoside, Methicillin, β-lactam, Fusidic acid	0	1 (7)
**7**	Aminoglycoside, Methicillin, β-lactam, Fluoroquinolone, Fusidic acid, Lincosamide, Macrolide, Trimethoprim-sulfamethoxazole	0	2 (14)
**8**	Macrolide, β-lactam	0	1 (7)
**9**	Methicillin, β-lactam	0	1 (7)
**10**	Aminoglycoside, Methicillin, β-lactam, Fluoroquinolone, Lincosamide, Macrolide, Trimethoprim-sulfamethoxazole	0	1 (7)
**11**	β-lactam	0	1 (7)

Combination	Antibiotic resistance genotype	Outbreak clone n (%)	Non-outbreak isolates n (%)
**1**	*aac(6')-aph(2"), blaZ, qacA, mphC, msrA, mecA*	15 (75)	4 (29)
**2**	*aac(6')-aph(2"), blaZ, mphC, msrA, mecA*	3 (15)	0
**3**	*aac(6')-aph(2"), blaZ, qacA, mecA*	2 (10)	0
**4**	*blaZ, qacA*	0 (0)	1 (7)
**5**	*aadD, blaZ, cat (pC221), qacA, fusB*	0	1 (7)
**6**	*aac(6')-aph(2"), blaZ, mecA, tet*(M)	0	1 (7)
**7**	*aac(6')-aph(2"), blaZ, cat (pC221). qacA, fusB, lsaA, mphC, vga(A)LC, msrA, mecA, tet*(K), *dfrG*	0	2 (14)
**8**	*aac(6')-aph(2"), blaZ, cat (pC221), qacA, mecA, tet*(K)	0	1 (7)
**9**	*aph(3')-III*	0	1 (7)
**10**	*blaZ, qacA, mecA, tet*(K)	0	1 (7)
**11**	*aac(6')-aph(2"), blaZ, qacA, fusB, fusc, mecA*	0	1 (7)
**12**	*aac(6')-aph(2"), blaZ, qacA, ermC, mphC, msrA, mecA, dfrG*	0	1 (7)

**Combination**	**Virulence**	**Outbreak clone n (%)**	**Non-outbreak isolates n (%)**
**1**	All 20 virulence genes (Table S1)	20 (100)	2 (14)
**2**	All virulence genes except *SH2607_NA* (Table S1)	0	5 (36)
**3**	All virulence genes except *folB* (Table S1)	0	1 (7)
**4**	*SH0155_NA, nikA, yjjG, ansA, blaZ, SH2156_NA*	0	1 (7)
**5**	*SH0155_NA, nikA, secA2, fabG, yjjG, ansA, blaI, blaR1, blaZ, SH2156_NA, tagE, sraP*	0	2 (14)
**6**	*ugpQ, paaZ, mecA, SH0155_NA, nikA, secA2, fabG, yjjG, ansA, aacA-aphD, SH1612_NA, blaI, blaR1, blaZ, SH2156_NA, tagE, sraP*	0	1 (7)
**7**	*ugpQ, paaZ, mecA, SH0155_NA, nikA, secA2, fabG, yjjG, ansA, aacA-aphD, SH1612_NA, blaI, blaZ, tagE, folB, folP, SH2607_NA, sraP*	0	1 (7)
**8**	*ugpQ, paaZ, mecA, SH0155_NA, nikA, secA2, fabG, yjjG, ansA, blaI, blaR1, blaZ, SH2156_NA, tagE, sraP*	0	1 (7)

Supplementary reference

1. Pain M, Hjerde E, Klingenberg C, Cavanagh JP. 2019. Comparative Genomic Analysis of Staphylococcus haemolyticus Reveals Key to Hospital Adaptation and Pathogenicity. Front Microbiol 10:2096.
